# Genome-wide association study reveals two loci for serum magnesium concentrations in European-American children

**DOI:** 10.1038/srep18792

**Published:** 2015-12-21

**Authors:** Xiao Chang, Joseph Glessner, Adrienne Tin, Jin Li, Yiran Guo, Zhi Wei, Yichuan Liu, Frank D. Mentch, Cuiping Hou, Yan Zhao, Tiancheng Wang, Haijun Qiu, Cecilia Kim, Patrick M. A. Sleiman, Hakon Hakonarson

**Affiliations:** 1 The Center for Applied Genomics, Children’s Hospital of Philadelphia, Philadelphia, Pennsylvania, 19104, USA; 2 Department of Epidemiology, Johns Hopkins Bloomberg School of Public Health, Baltimore, Maryland, 21205, USA; 3 Department of Computer Science, New Jersey Institute of Technology, Newark, New Jersey, 07102, USA; 4 Department of Pediatrics, The Perelman School of Medicine, University of Pennsylvania, Philadelphia, Pennsylvania, 19104, USA; 5 Division of Human Genetics, Children’s Hospital of Philadelphia, Philadelphia, Pennsylvania, 19104, USA

## Abstract

Magnesium ions are essential to the basic metabolic processes in the human body. Previous genetic studies indicate that serum magnesium levels are highly heritable, and a few genetic loci have been reported involving regulation of serum magnesium in adults. In this study, we examined if additional loci influence serum magnesium levels in children. We performed a genome-wide association study (GWAS) on 2,267 European-American children genotyped on the Illumina HumanHap550 or Quad610 arrays, sharing over 500,000 markers, as the discovery cohort and 257 European-American children genotyped on the Illumina Human OmniExpress arrays as the replication cohort. After genotype imputation, the strongest associations uncovered were with imputed SNPs residing within the *FGFR2* (rs1219515, *P* = 1.1 × 10^−5^) and *PAPSS2* (rs1969821, *P* = 7.2 × 10^−6^) loci in the discovery cohort, both of which were robustly replicated in our independent patient cohort (rs1219515, *P* = 3.5 × 10^−3^; rs1969821, *P* = 1.2 × 10^−2^). The associations at the *FGFR2* locus were also weakly replicated in a dataset from a previous GWAS of serum magnesium in European adults. Our results indicate that *FGFR2* and *PAPSS2* may play an important role in the regulation of magnesium homeostasis in children of European-American ancestry.

Magnesium is an essential cation, which plays an important role in multiple physiological functions. It has been reported that magnesium deficiency is common in the general population and low serum magnesium levels are associated with a variety of diseases including Type 2 diabetes mellitus, hypertension and osteoporosis^1^,^2^,^3^,^4^. Currently, serum magnesium levels are frequently measured to assess for deficiency in magnesium status in relation with various medical symptoms and complaints.

Although low dietary intake is the major cause of magnesium deficiency, several studies demonstrate that differences in serum magnesium levels can be explained in part by genetic variations with the heritability estimates ranging from 19% to 39%^5^,^6^. In addition, a GWA study has identified common variants at six loci (*MUC1, ATP2B1, DCDC5, TRPM6, SHROOM3*, and *MDS1*), influencing serum magnesium levels of European adults^7^. Moreover, our recent GWAS successfully replicated the *MUC1* locus in European-American children^8^. Considering that serum magnesium levels vary between children and adults^9^, we hypothesize that certain common variants may specifically influence serum magnesium levels in children during their rapid growth and development phases^4^,^10^.

In this study, we have increased the sample size of our previous cohort genotyped on the Illumina HumanHap550 or Quad610 arrays, and further identified an independent cohort genotyped on the Illumina HumanOmniExpress arrays, aiming to uncover and replicate genomic loci influencing serum magnesium levels in children.

## Results

**Figure 1:**
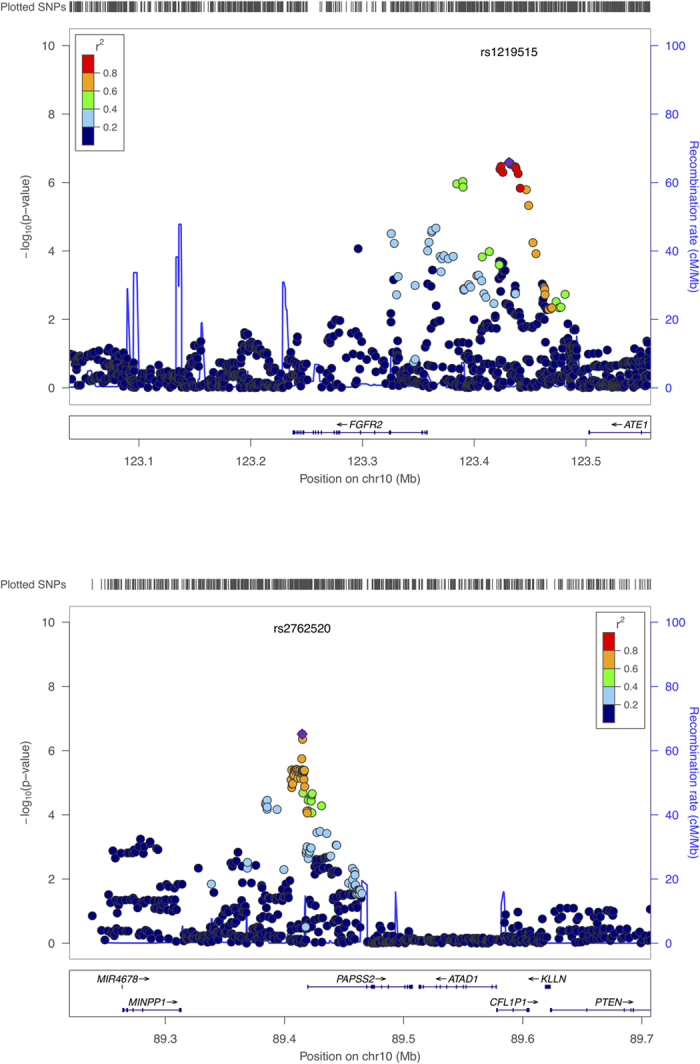
Regional association plots of the imputed SNPs at *FGFR2* and *PAPSS2* SNPs are plotted as the −log10 of the p-value (meta-analysis of combined discovery and replication cohorts). The top SNP was colored in purple. The correlation (r^2^) is estimated in the EUR population from the 1000 Genomes Project (Mar 2012). The plot was generated by LocusZoom^26^.

The discovery cohort consisted of 2,267 European-American children genotyped on the Illumina HumanHap550 or Quad610 arrays. Beside the previously discovered *MUC1* locus, two additional loci were nominally associated with serum magnesium concentrations (*FGFR2*, rs2935713, *P* = 3.6 × 10^−5^; *PAPSS2*, rs791888, *P* = 1.6 × 10^−5^; Supplementary Table 1). Although neither of these loci reaches genome-wide significance, mutations in either *FGFR2* or *PAPSS2* have been reported to result in different types of bone diseases such as skeletal dysplasia and craniosynostosis^11^,^12^,^13^,^14^,^15^. We therefore identified a replication cohort of 257 European-American children genotyped on the Illumina HumanOmniExpress arrays. Both the *FGFR2* and *PAPSS2* loci were validated in the replication cohort (*FGFR2*, rs2935713, *P* = 1.5 × 10^−3^; *PAPSS2*, rs791888, *P* = 0.04; Supplementary Table 1). To identify additional SNPs not assayed directly on the SNP arrays, we imputed unobserved SNPs at *FGFR2* and *PAPSS2* using 1000 Genomes Project data as the reference. Meta-analysis of the imputed SNPs from both discovery and replication cohorts identified 16 SNPs at the *FGFR2* locus (The most significant SNP is rs1219515, *P* = 2.6 × 10^−7^) and 30 SNPs at the *PAPSS2* locus (The most significant SNP is rs2762520, *P* = 3.1 × 10^−7^) that were associated with serum magnesium concentrations (*P* < 1 × 10^−5^, Table 1 and Supplementary Table 2). The top SNPs from *FGFR2* locus resided in a strong linkage disequilibrium region (r2 > 0.8) near *FGFR2*, while the top SNPs from the *PAPSS2* locus resided in a strong linkage disequilibrium region (r2 > 0.6) within *PAPSS2* (Fig. 1).

We further evaluated the lead SNPs within the *FGFR2* and *PAPSS2* loci in a previous meta-analysis of 15,366 adults of European descent from three independent cohorts (the Atherosclerosis Risk in Communities study (ARIC), the Framingham Heart Study (FHS) and the Rotterdam Study (RS))^7^. The best proxies of the lead SNPs were used, if the lead SNPs were not available in the adult meta-analysis. The lead imputed SNPs of meta-analysis, rs1219515 and rs2762520 in *FGFR2* and *PAPSS2*, are not available in the adult study. However, for *FGFR2* gene locus, rs2935713 (*P* = 2.7 × 10^−7^ in the meta-analysis of children study), the best proxy of rs1219515 (*r*^2^ = 0.89) available in the adult cohort, has a *P* = 0.036 (*P* value adjusted by Bonferroni correction is 0.072) in the meta-analysis of adult study. For *PAPSS2* gene locus, rs791885 (*P* = 4.0 × 10^−7^ in the meta-analysis of children study), the best proxy of rs2762520 (*r*^2^ = 0.63) available in the adult cohort, has a *P* = 0.38 in the meta-analysis of adult study (Supplementary Table 3). Though the *PAPSS2* locus is not significant in the meta-analysis of the adult study, rs791885 is significant in the RS cohort with a *P* = 3.6 × 10^−3^ (*P* value adjusted by Bonferroni correction is 0.0072) (Supplementary Table 3).

## Discussion

We identified two loci associated with serum magnesium levels in European-American children, which target genes previously reported in a broad spectrum of bone disorders. The product of *FGFR2* is a member of the fibroblast growth factor receptor family, which plays an essential role in osteoblast differentiation and normal skeleton development. Defects of *FGFR2* can result in Mendelian diseases involving abnormal bone development, such as Crouzon syndrome [MIM:123500], Jackson-Weiss syndrome [MIM:123150], Apert syndrome [MIM:101200], Pfeiffer syndrome [MIM:101600] and Bent bone dysplasia syndrome [MIM:614592]^11^,^12^,^15^. *PAPSS2* encodes the enzyme Bifunctional 3′-phosphoadenosine 5′-phosphosulfate synthetase 2. Mutations in *PAPSS2* have been reported to result in osteochondrodysplasias such as Spondyloepimetaphyseal dysplasia Pakistani type [MIM:612847]^13^,^14^,^16^. Given that magnesium is crucial to bone formation and development, *FGFR2* and *PAPSS2* may influence the level of serum magnesium through bone metabolism^4^,^17^.

We also examined the associations of the *FGFR2* and *PAPSS2* loci in three cohorts of adults. The top SNP rs2935713 from the *FGFR2* locus was nominally associated with serum magnesium levels in in a meta-analysis of three Caucasian cohorts. The association of top SNP rs791885 within *PAPSS2* was supported by the RS cohort. However, the associations of both loci in adults are much weaker than those in children. Though common SNPs were investigated in this study, potential bias could exist if the minor allele frequencies are significantly different from children and adult cohort in this study. To address this question, we have checked the minor allele frequencies of the lead imputed SNPs of *FGFR2* and *PAPSS2* loci in the children and adult cohorts, and the MAFs are very similar among all the cohorts (Supplementary Table 3). As serum magnesium levels were associated with hypertension, diabetes, and osteoporosis in previous studies, we further re-analyzed our discovery cohort stratified by disease groups (individuals with hypertension, diabetes or osteoporosis; individuals without any conditions of hypertension, diabetes or osteoporosis), and the *FGFR2* and *PAPSS2* loci were significant in both groups (Supplementary Table 4). Taken together, these results suggest that *FGFR2* and *PAPSS2* influence serum magnesium levels of individuals in their childhood. As described above, *FGFR2* and *PAPSS2* are associated with various diseases involving bone development. Therefore, these genes may primarily participate in the bone metabolism of children during their growth, and influence serum magnesium levels through that mechanism in children.

In this study, we have uncovered two loci associated with serum magnesium levels in children. Our results suggested that *FGFR2* and *PAPSS2* may influence the regulation of magnesium homeostasis in children. Future GWA studies with larger sample sizes are likely to identify additional loci for serum magnesium, and follow-up functional studies of *FGFR2* and *PAPSS2* may further delineate their role in the regulation of magnesium homeostasis in children.

## Materials and Methods

### Ethics statement

This study was approved by the Research Ethics Board at the Children’s Hospital of Philadelphia (CHOP) and carried out in accordance with the nationally approved guidelines. Written informed consent was obtained from all research subjects and/or their parents by nursing and medical assistant staff under the direction of CHOP clinicians.

### Sample collection

A total of 4,898 patients were recruited from The Children’s Hospital of Philadelphia (CHOP) Health Care Network. Among them, 4,473 were genotyped on the Illumina HumanHap550 or Quad610 arrays, and 425 were genotyped on the Illumina HumanOmniExpress arrays. The genotyping procedure follows the Illumina standard protocols as previously described^18^. European-American children were genetically inferred by EIGENSTRAT^19^ (Fig. S1). After quality control, 2,267 samples genotyped on the Illumina HumanHap550 or Quad610 arrays were included as the discovery cohort, and 257 samples genotyped on the Illumina HumanOmniExpress arrays were included as the replication cohort. The values of serum magnesium levels displayed normal distribution in both discovery and replication cohorts (Fig. S2). Subjects with values beyond 3SD of the mean were removed from the study. Details of the discovery and replication cohorts used in this study are summarized in (Supplementary Table 5).

### Quality control

Subjects with genotype call rate below 95% were excluded from the study. GCTA was used to estimated cryptic relatedness between samples, one of a pair of individuals was removed if the estimated relatedness score was larger than 0.025^20^. SNPs with genotype missing rate <5%, minor allele frequency >1%, and Hardy-Weinberg equilibrium P value < 0.00001 were included. 507,409 and 619,523 markers were included for the association test of serum magnesium levels in the discovery and replication cohorts, respectively.

### Statistical analysis

The association analyses were carried out in PLINK using linear regression models^21^. The additive model was used for the association test. The meta-analyses were performed by METAL^22^. The fixed effects model was used for the meta-analysis. Age, gender and the first ten PCs calculated by EIGENSTRAT were included as covariates. The genomic inflation factors was 1.0 for the analysis performed (Fig. S3).

Genotype imputation at the *FGFR2* (genomic region chr8: 123,000,000-123,600,000; hg19) and *PAPSS2* (genomic region chr8: 89,200,000-89,800,000; hg19) gene loci was performed with IMPUTE2 using the reference panel 1000 Genome Phase I integrated variants set (Dec 2012 release)^23^. SHAPEIT recommended by Howie *et al.* was used to infer the haplotypes before imputation^24^. In consideration of the uncertainty of imputation, the association test of the imputed genotypes was calculated with the SNPTEST v2 package^25^. Imputed SNPs with info score >0.9, minor allele frequency >1%, and Hardy-Weinberg equilibrium P value < 0.00001 were included. For *FGFR2*, 1,516 and 1,681 imputed SNPs were included for the association test in discovery and replication cohorts respectively. The 1,486 shared SNPs between discovery and replication were used for the meta-analysis. For *PAPSS2*, 1,107 and 1,214 imputed SNPs were included for the association test in discovery and replication cohorts respectively. The 1,065 shared SNPs between discovery and replication were used for the meta-analysis.

### Replication cohorts from adult samples

A total of 15,366 adults of European descent from three independent cohorts (ARIC, FHS and RS) in a previously reported study were used as replications^7^. Details of samples collection and statistic analysis for those data can be found elsewhere^7^.

## Additional Information

**How to cite this article**: Chang, X. *et al.* Genome-wide association study reveals two loci for serum magnesium concentrations in European-American children. *Sci. Rep.* **5**, 18792; doi: 10.1038/srep18792 (2015).

## Supplementary Material

Supplementary Information

## Figures and Tables

**Table 1:** Associations between serum magnesium levels and the lead imputed SNPs in the discovery analysis.

Gene		*PAPSS2*	*FGFR2*
**SNP**		rs1969821	rs1219515
**POS**		89415148	123431245
**A1**		A	A
**A2**		G	G
**Discovery**	**BETA**	2.8E-02	−4.8E-02
	**SE**	6.2E-03	1.1E-02
	**P**	7.2E-06	1.1E-05
**Replication**	**BETA**	4.6E-02	−8.2E-02
	**SE**	1.8E-02	2.8E-02
	**P**	1.2E-02	3.5E-03
**P-adj**	3.7E-02	1.0E-02	
**Combined**	**BETA**	3.0E-02	−5.3E-02
	**SE**	5.9E-03	1.0E-02
	**P**	4.4E-07	2.6E-07

A1: Coded allele.

A2: Uncoded allele.

Beta: Regression coefficient (unit, mg/dl).

SE: Standard error of Beta.

P-adj: P-values adjusted by Bonferroni correction (based on the three examined SNPs in replication).
