# Supplementary material for: Genome-wide association study reveals two loci for serum magnesium concentrations in European-American children
Source: Sci Rep. 2015 Dec 21;5:18792. doi: 10.1038/srep18792 (PMC4685389; doi:10.1038/srep18792)
Supplement: Supplementary Information [file srep18792-s1.pdf]

Genome-wide association study reveals two loci for serum magnesium concentrations in European-American children

Xiao Chang[1], Joseph Glessner[1], Adrienne Tin[2], Jin Li[1], Yiran Guo[1], Zhi Wei[3], Yichuan Liu[1], Frank D Mentch[1], Cuiping Hou[1], Yan Zhao[1], Tiancheng Wang[1], Haijun Qiu[1], Cecilia Kim[1], Patrick M A Sleiman[1, 4, 5], Hakon Hakonarson[1, 4, 5, *]

[1]The Center for Applied Genomics, Children's Hospital of Philadelphia, Philadelphia, Pennsylvania, 19104, USA.

[2]Department of Epidemiology, Johns Hopkins Bloomberg School of Public Health, Baltimore, Maryland, 21205, USA

[3]Department of Computer Science, New Jersey Institute of Technology, Newark, New Jersey, 07102, USA.

[4]Department of Pediatrics, The Perelman School of Medicine, University of Pennsylvania, Philadelphia, Pennsylvania, 19104, USA.

[5]Division of Human Genetics, Children's Hospital of Philadelphia, Philadelphia, Pennsylvania, 19104, USA.

[*]Correspondence should be addressed to H.H. (hakonarson@email.chop.edu)

Supplementary Figure 1. Plot of HapMap3 populations and subjects used in this study. The first two PCs were plotted.

a. Plot of the discovery cohort genotyped on the Illumina HumanHap550 or Quad610 arrays.

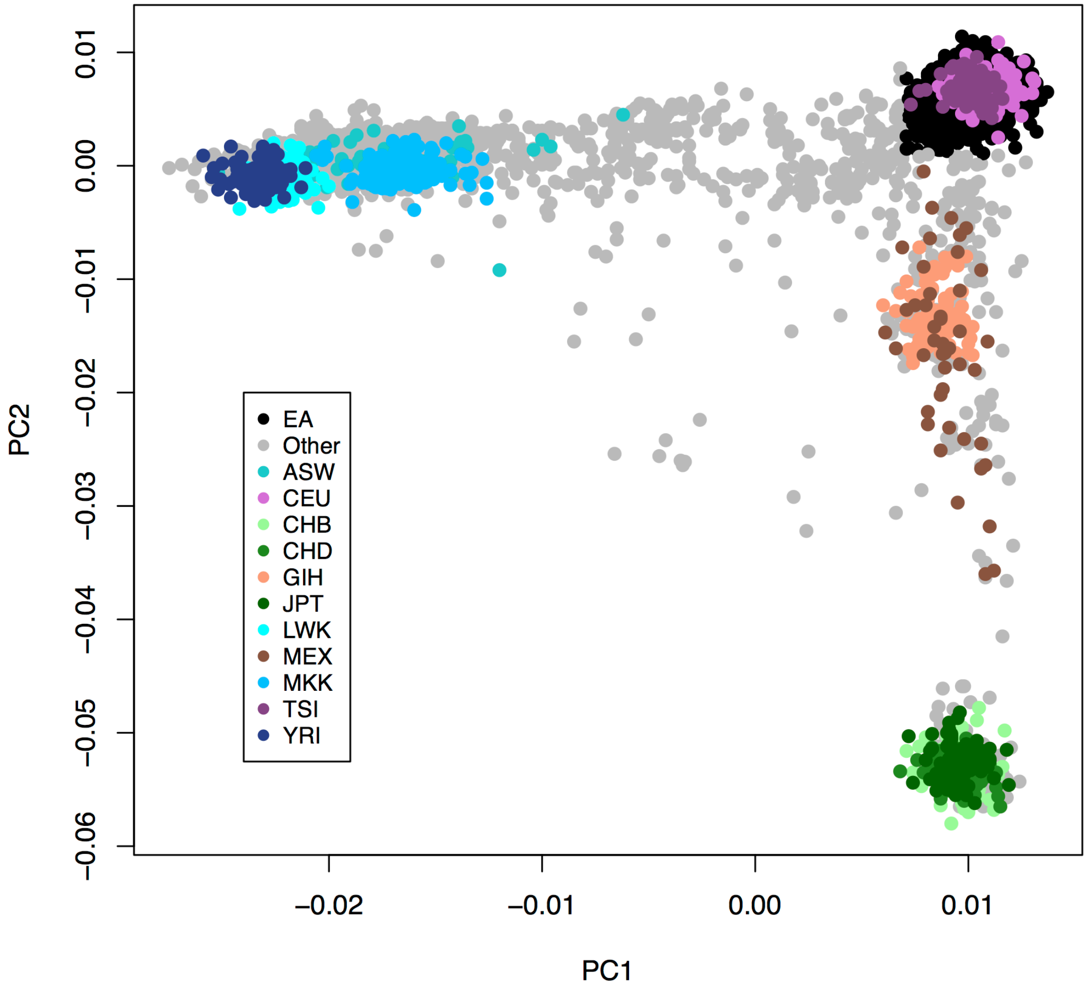

b. Plot of the replication cohort genotyped on the Illumina Human Omni-Express arrays.

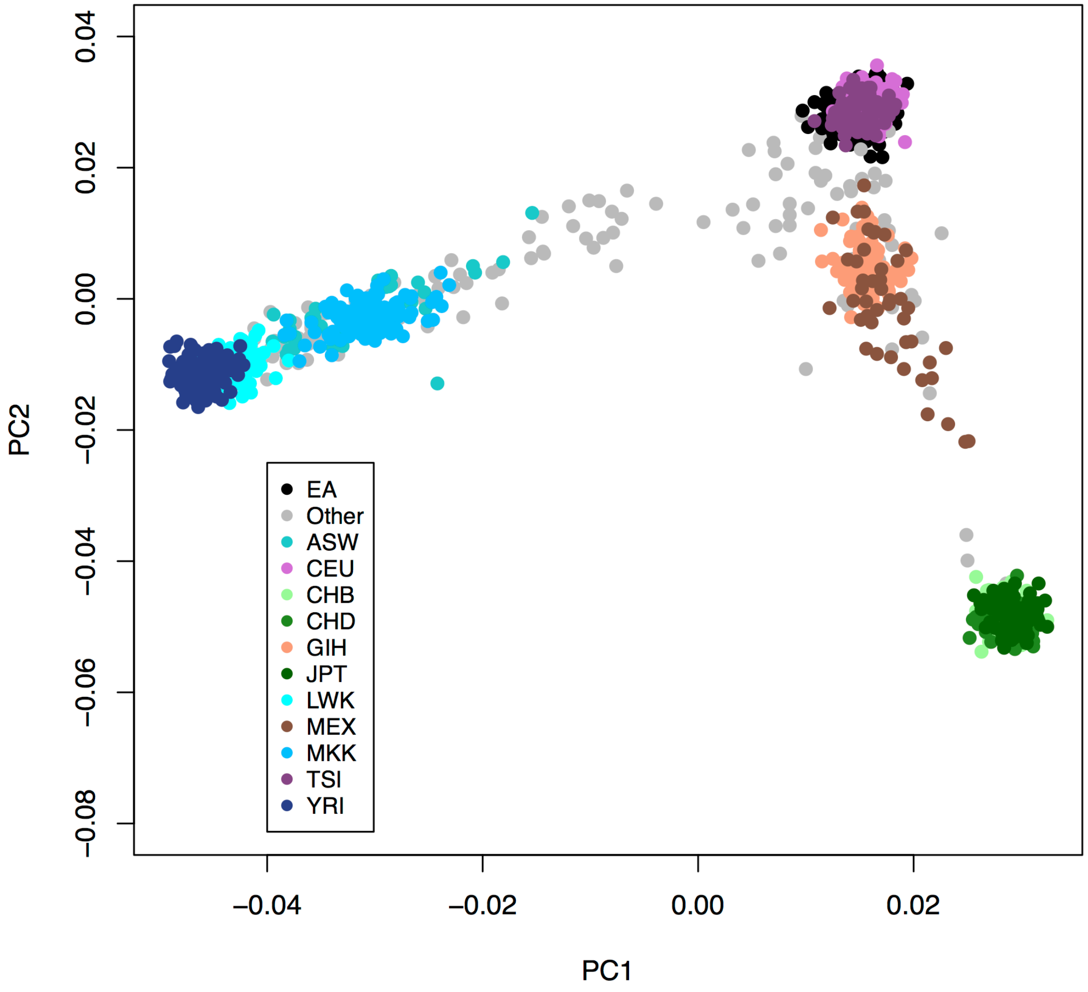

Supplementary Figure 2. Distributions of serum magnesium levels in the discovery and replication cohorts.

a. Discovery cohort.

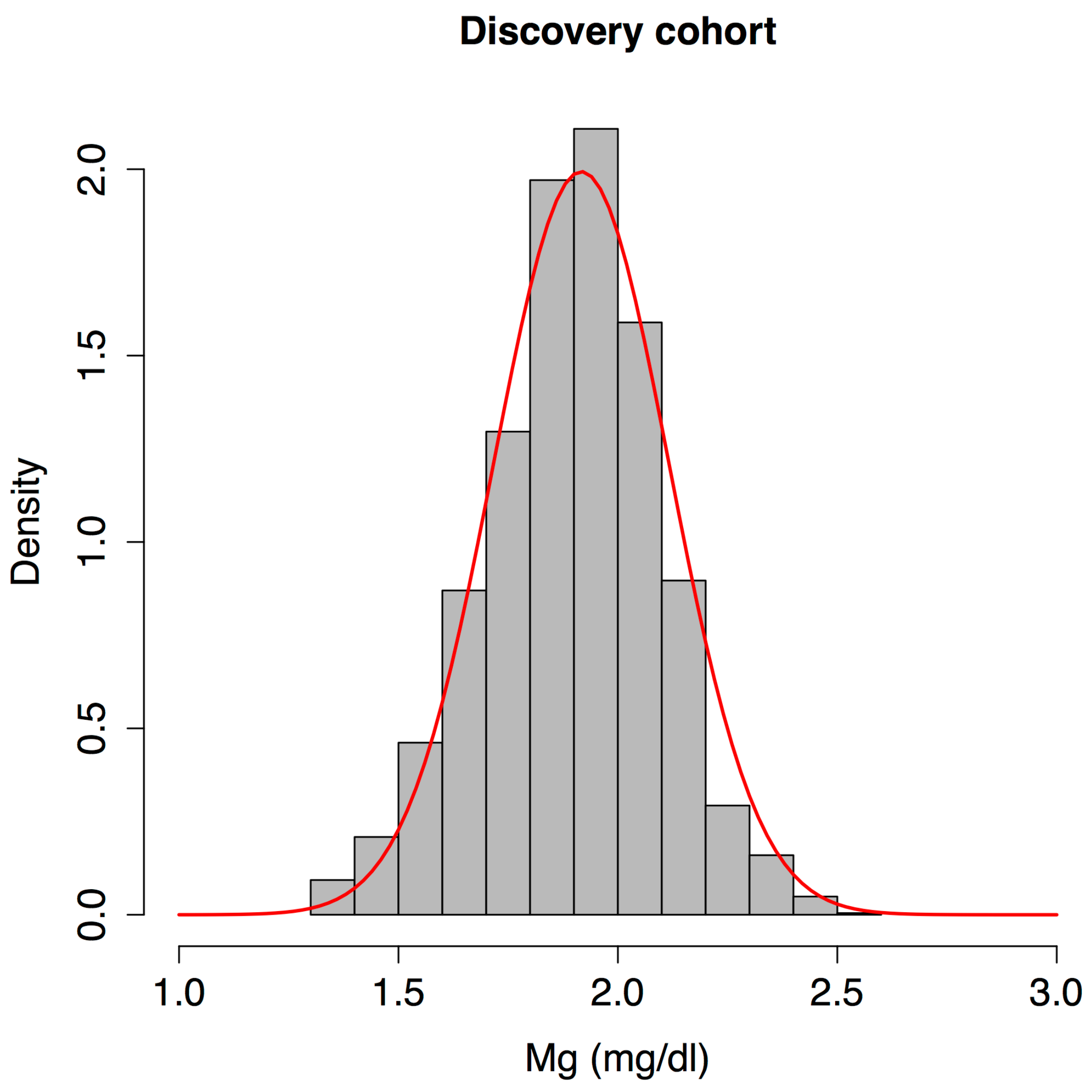

b. Replication cohort.

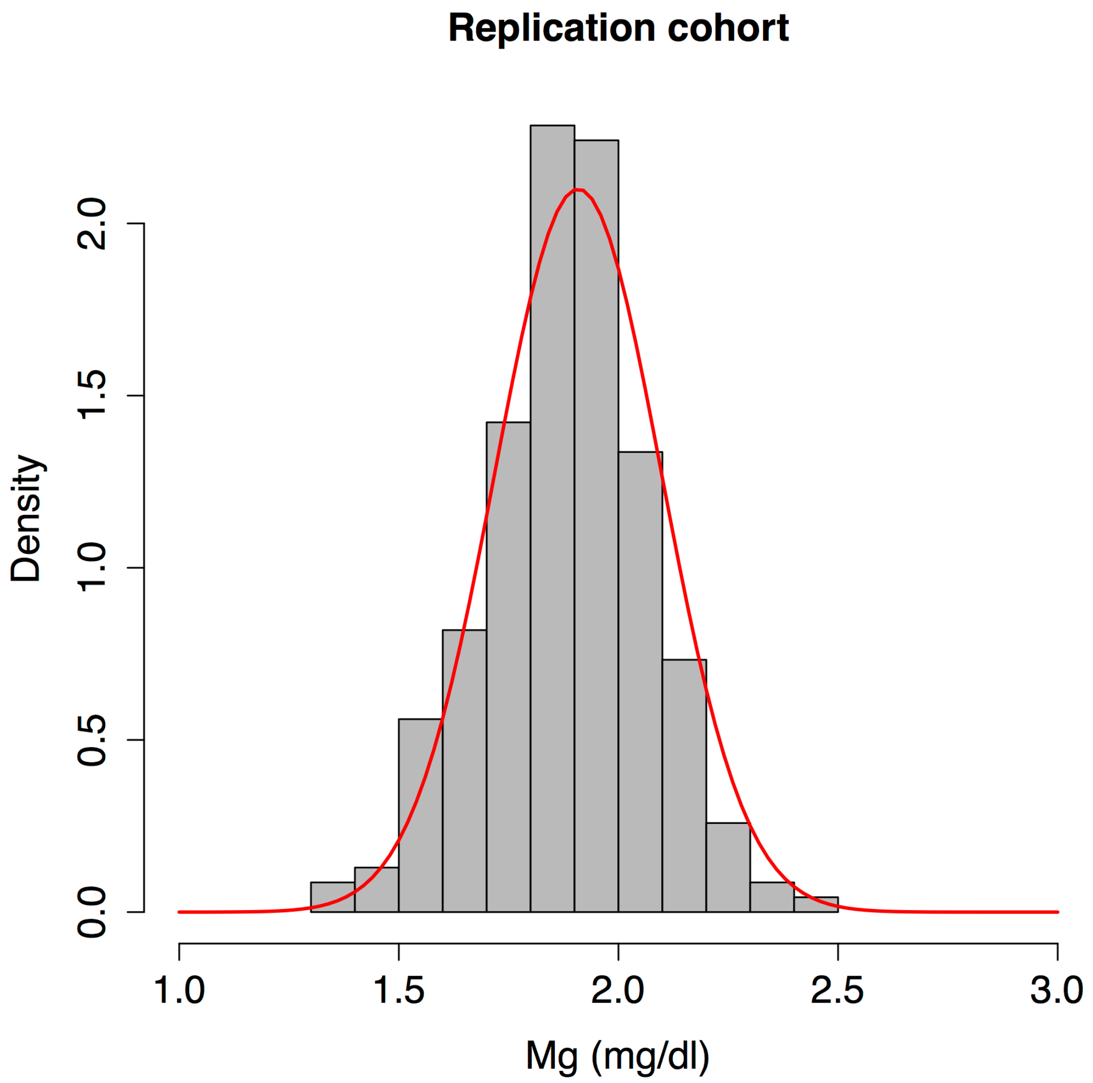

Supplementary Figure 3. Quantile-Quantile plot of the expected and observed P-values.

a. SNPs passing quality control in the discovery GWAS are plotted.

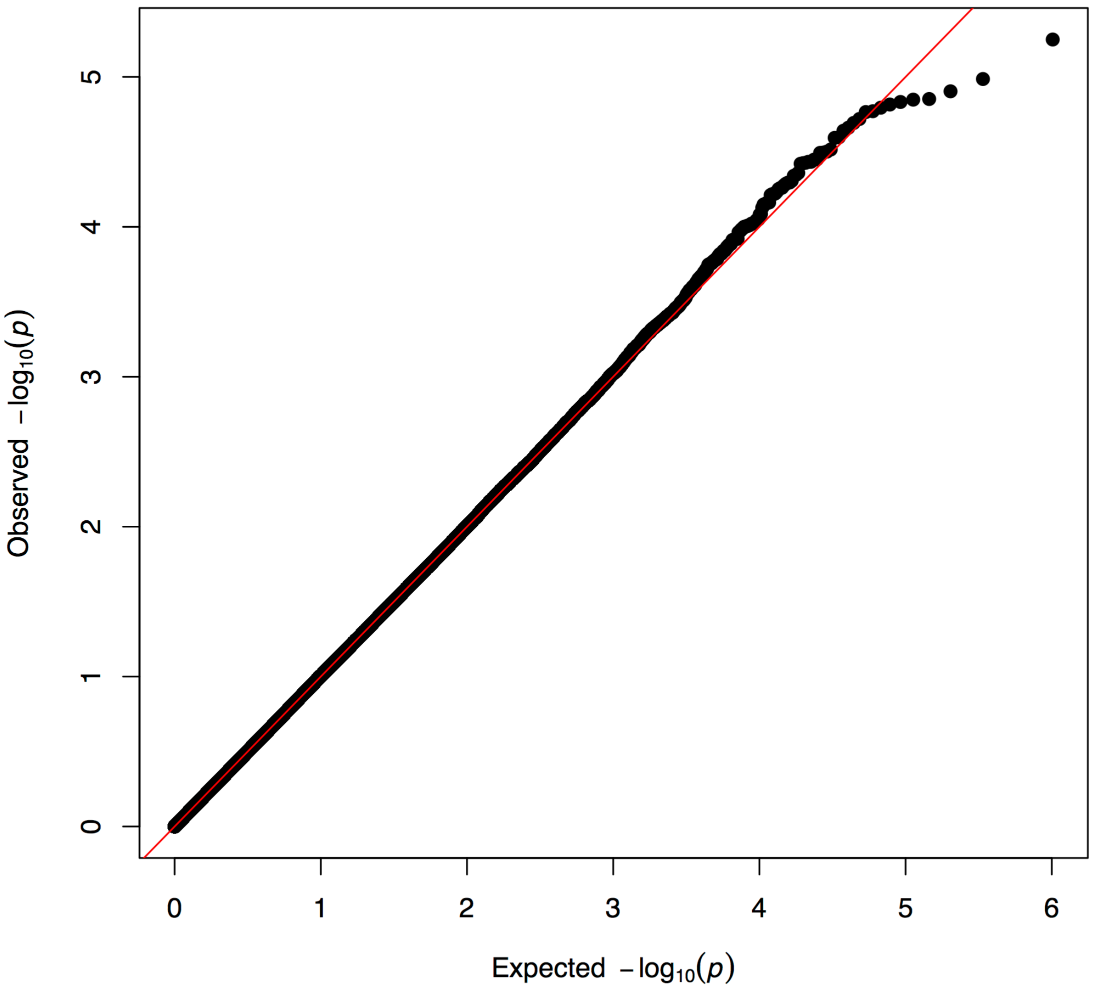

b. SNPs passing quality control in the replication GWAS are plotted.

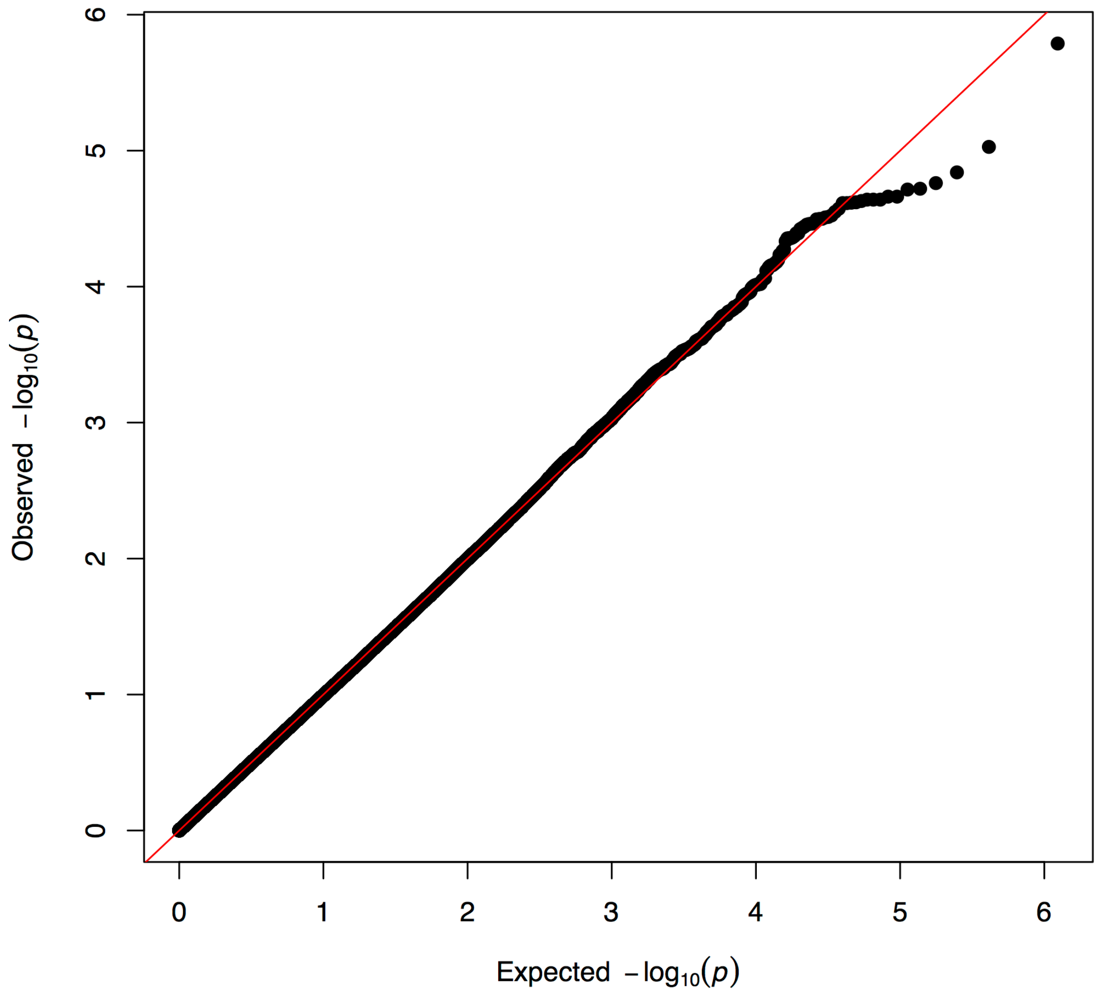

Supplementary Table 1. Associations between serum magnesium levels and the lead genotyped SNPs in the discovery analysis.

Gene	SNP	POS	A1	Discovery			Replication				Combined		
				BETA	SE	P	BETA	SE	P	P-adj	BETA	SE	P
PAPSS2	rs791888	89412575	T	2.6E-02	6.1E-03	1.6E-05	3.9E-02	1.9E-02	4.0E-02	1.2E-01	2.7E-02	5.8E-03	2.8E-06
FGFR2	rs2935713	123432188	A	-4.7E-02	1.1E-02	3.8E-05	-9.3E-02	2.9E-02	1.5E-03	4.5E-03	-5.3E-02	1.0E-02	2.9E-07

A1: Coded allele

Beta: Regression coefficient (unit, mg/dl)

SE: Standard error of BETA

P-adj: P-values adjusted by Bonferroni correction (based on the three examined SNPs in replication)

Supplementary Table 2. Associations between serum magnesium levels and the imputed SNPs residing at the *FGFR2* and *PAPSS2* loci in the discovery, replication and meta-analysis (imputed SNPs with P values < 10^{-5} in the meta-analysis were included).

Gene	SNP	POS	A1	A2	Discovery			Replication			Combined		
					**BETA**	**SE**	**P**	**BETA**	**SE**	**P**	**BETA**	**SE**	**P**
	rs2762524	89416693	G	A	2.5E-02	6.1E-03	5.4E-05	4.4E-02	1.8E-02	1.5E-02	2.7E-02	5.8E-03	4.1E-06
	rs2762523	89416650	C	T	2.5E-02	6.1E-03	5.2E-05	4.4E-02	1.8E-02	1.5E-02	2.7E-02	5.8E-03	4.1E-06
	rs2755429	89416464	A	C	2.5E-02	6.1E-03	5.3E-05	4.3E-02	1.8E-02	1.7E-02	2.7E-02	5.8E-03	4.3E-06
	rs2180970	89416350	T	G	2.5E-02	6.0E-03	3.4E-05	3.8E-02	1.8E-02	3.9E-02	2.6E-02	5.7E-03	4.6E-06
	rs1609579	89416342	A	G	2.4E-02	6.1E-03	8.2E-05	4.1E-02	1.8E-02	2.2E-02	2.6E-02	5.8E-03	8.0E-06
	rs2147288	89415882	A	C	2.5E-02	6.0E-03	3.3E-05	3.7E-02	1.8E-02	4.3E-02	2.6E-02	5.7E-03	4.7E-06
	rs2038680	89415755	G	A	2.5E-02	6.1E-03	4.8E-05	4.2E-02	1.8E-02	1.9E-02	2.7E-02	5.8E-03	4.2E-06
	rs2755430	89415605	T	C	2.5E-02	6.1E-03	4.7E-05	4.2E-02	1.8E-02	2.0E-02	2.7E-02	5.8E-03	4.1E-06
	rs2762522	89415541	G	A	2.4E-02	6.1E-03	6.1E-05	4.2E-02	1.8E-02	2.0E-02	2.6E-02	5.8E-03	5.4E-06
	rs1969821	89415148	A	G	2.8E-02	6.2E-03	7.2E-06	4.6E-02	1.8E-02	1.2E-02	3.0E-02	5.9E-03	4.4E-07
	rs2755431	89415104	T	C	2.5E-02	6.1E-03	6.0E-05	4.7E-02	1.8E-02	1.1E-02	2.7E-02	5.8E-03	4.0E-06
	rs2762521	89414923	A	T	2.5E-02	6.0E-03	3.4E-05	3.7E-02	1.8E-02	4.5E-02	2.6E-02	5.7E-03	5.0E-06
	rs2762520	89414845	G	A	2.6E-02	5.9E-03	8.4E-06	5.0E-02	1.8E-02	4.8E-03	2.9E-02	5.6E-03	3.1E-07
	rs2755432	89414779	T	C	2.5E-02	6.1E-03	4.7E-05	4.2E-02	1.8E-02	2.0E-02	2.7E-02	5.8E-03	4.2E-06
PAPSS2	rs2762519	89414479	C	T	2.6E-02	6.1E-03	2.3E-05	4.4E-02	1.8E-02	1.6E-02	2.8E-02	5.8E-03	1.8E-06
	rs1408378	89413960	A	G	2.5E-02	6.1E-03	5.1E-05	4.1E-02	1.8E-02	2.3E-02	2.6E-02	5.8E-03	5.0E-06
	rs1830588	89413479	A	G	2.4E-02	6.0E-03	4.9E-05	3.7E-02	1.8E-02	4.5E-02	2.6E-02	5.7E-03	7.3E-06
	rs791889	89413057	T	C	2.5E-02	6.1E-03	4.7E-05	4.2E-02	1.8E-02	2.0E-02	2.7E-02	5.8E-03	4.1E-06
	rs791888	89412575	T	G	2.5E-02	6.0E-03	3.3E-05	3.7E-02	1.8E-02	4.5E-02	2.6E-02	5.7E-03	4.9E-06
	rs791887	89411554	C	T	2.5E-02	6.1E-03	6.0E-05	4.6E-02	1.8E-02	1.2E-02	2.7E-02	5.8E-03	4.3E-06
	rs791886	89411315	C	T	2.4E-02	6.0E-03	4.9E-05	3.7E-02	1.8E-02	4.5E-02	2.6E-02	5.7E-03	7.3E-06
	rs791885	89410546	A	T	2.5E-02	6.1E-03	4.5E-05	4.2E-02	1.8E-02	2.0E-02	2.7E-02	5.8E-03	3.9E-06
	rs791884	89410076	C	T	2.5E-02	6.1E-03	4.4E-05	4.2E-02	1.8E-02	2.0E-02	2.7E-02	5.8E-03	3.8E-06
	rs791883	89409287	T	A	2.5E-02	6.1E-03	4.9E-05	4.2E-02	1.8E-02	2.0E-02	2.7E-02	5.8E-03	4.3E-06
	rs2755434	89409027	G	C	2.5E-02	6.1E-03	4.7E-05	4.2E-02	1.8E-02	2.0E-02	2.7E-02	5.8E-03	4.2E-06
	rs791882	89408258	T	C	2.5E-02	6.0E-03	3.9E-05	3.7E-02	1.8E-02	4.5E-02	2.6E-02	5.7E-03	5.7E-06
	rs791881	89408246	T	C	2.5E-02	6.1E-03	4.6E-05	4.2E-02	1.8E-02	2.0E-02	2.7E-02	5.8E-03	4.1E-06
	rs791878	89407378	G	C	2.5E-02	6.0E-03	4.1E-05	3.7E-02	1.8E-02	4.5E-02	2.6E-02	5.7E-03	6.1E-06
	rs791874	89406070	G	T	2.5E-02	6.1E-03	4.5E-05	4.2E-02	1.8E-02	2.0E-02	2.7E-02	5.8E-03	4.0E-06
	rs791873	89405558	T	C	2.4E-02	6.0E-03	5.5E-05	3.7E-02	1.8E-02	4.4E-02	2.6E-02	5.7E-03	8.2E-06
FGFR2	rs11598352	123384389	A	G	-5.5E-02	1.3E-02	1.5E-05	-8.2E-02	3.5E-02	1.8E-02	-5.8E-02	1.2E-02	1.1E-06
	rs117419410	123389837	A	G	-5.5E-02	1.3E-02	1.4E-05	-8.0E-02	3.4E-02	1.8E-02	-5.8E-02	1.2E-02	9.4E-07

	rs75752394	123390024	A	G	-5.4E-02	1.3E-02	2.1E-05	-8.3E-02	3.4E-02	1.5E-02	-5.8E-02	1.2E-02	1.4E-06
	rs4751846	123423047	A	G	-4.7E-02	1.1E-02	1.7E-05	-8.2E-02	2.8E-02	3.5E-03	-5.2E-02	1.0E-02	4.1E-07
	rs11594647	123423971	C	T	-4.7E-02	1.1E-02	1.7E-05	-8.3E-02	2.8E-02	2.8E-03	-5.2E-02	1.0E-02	3.4E-07
	rs77180105	123425657	G	A	-4.8E-02	1.1E-02	1.5E-05	-7.7E-02	2.8E-02	6.5E-03	-5.2E-02	1.0E-02	5.1E-07
	rs1219515	123431245	A	G	-4.8E-02	1.1E-02	1.1E-05	-8.2E-02	2.8E-02	3.5E-03	-5.3E-02	1.0E-02	2.6E-07
	rs2935713	123432188	A	G	-4.8E-02	1.1E-02	1.9E-05	-9.0E-02	2.8E-02	1.5E-03	-5.4E-02	1.1E-02	2.7E-07
	rs77679651	123432575	C	G	-4.8E-02	1.1E-02	2.1E-05	-9.0E-02	2.8E-02	1.5E-03	-5.4E-02	1.1E-02	3.0E-07
	rs4752578	123436524	T	C	-4.8E-02	1.1E-02	2.3E-05	-9.0E-02	2.8E-02	1.6E-03	-5.4E-02	1.1E-02	3.4E-07
	rs78856329	123436834	T	C	-4.8E-02	1.1E-02	2.4E-05	-9.0E-02	2.8E-02	1.6E-03	-5.4E-02	1.1E-02	3.5E-07
	rs1875155	123437540	C	T	-4.7E-02	1.1E-02	2.6E-05	-9.0E-02	2.8E-02	1.6E-03	-5.3E-02	1.1E-02	3.9E-07
	rs77832491	123439319	A	G	-4.7E-02	1.1E-02	3.4E-05	-8.9E-02	2.8E-02	1.7E-03	-5.3E-02	1.1E-02	5.5E-07
	rs4752579	123441192	T	A	-4.5E-02	1.1E-02	7.2E-05	-8.6E-02	2.8E-02	2.3E-03	-5.0E-02	1.1E-02	1.5E-06
	rs79437746	123446630	T	G	-4.6E-02	1.1E-02	5.9E-05	-8.6E-02	3.0E-02	3.7E-03	-5.1E-02	1.1E-02	1.6E-06
	rs117971080	123448722	A	G	-4.4E-02	1.1E-02	1.1E-04	-8.1E-02	3.0E-02	7.4E-03	-4.9E-02	1.1E-02	4.7E-06

A1: Coded allele

A2: Uncoded allele

Beta: Regression coefficient (unit, mg/dl)

SE: Standard error of Beta

Supplementary Table 3. Associations between serum magnesium levels and the lead SNPs or the best proxy of the lead SNPs of the *FGFR2* and *PAPSS2* loci in three adult cohorts (ARIC, FHS and RS) and the current study (CHOP).

**Gene**		***PAPSS2***	***FGFR2***
**SNP**		rs791885	rs2935713
**CHR**		10	10
**POS**		89410546	123432188
**Meta-analysis of CHOP discovery and replication**	Minor_Allele	T	A
	MAF	4.0E-01	7.8E-02
	BETA	-2.7E-02	-5.4E-02
	SE	5.8E-03	1.1E-02
	P	3.9E-06	2.7E-07
**ARIC (n=8122)**	Minor_Allele	T	A
	MAF	4.0E-01	8.5E-02
	BETA	1.3E-02	-7.0E-02
	SE	2.1E-02	3.8E-02
	P	5.6E-01	6.2E-02
**FHS (n=2866)**	Minor_Allele	T	A
	MAF	4.1E-01	7.7E-02
	BETA	4.7E-03	-2.4E-02
	SE	4.2E-02	7.0E-02
	P	9.1E-01	7.3E-01
**RS (n=4378)**	Minor_Allele	T	A
	MAF	3.8E-01	8.9E-02
	BETA	-1.0E-01	-6.3E-02
	SE	3.5E-02	5.9E-02
	P	3.6E-03	2.8E-01
**Meta-analysis of ARIC, FHS and RS**	Minor_Allele	T	A
	MAF	4.0E-01	8.4E-02
	BETA	-1.4E-02	-6.1E-02
	SE	1.6E-02	2.9E-02
	P	3.8E-01	3.6E-02
	Direction	++-	---

MAF: Minor allele frequency

Beta: Regression coefficient (unit, mg/dl)

SE: Standard error of Beta

Supplementary Table 4. Associations between serum magnesium levels and the lead genotyped SNPs in the discovery cohort stratified by disease groups.

Gene	CHR	SNP	POS	A1	Group1 (n=1776)			Group2 (n=470)		
					BETA	SE	P	BETA	SE	P
PAPSS2	10	rs2755430	89415605	G	-2.6E-02	6.7E-03	1.1E-04	-2.9E-02	1.4E-02	3.6E-02
	10	rs791888	89412575	T	2.8E-02	6.6E-03	2.4E-05	2.7E-02	1.4E-02	5.3E-02
FGFR2	10	rs3135758	123277869	A	-5.2E-02	1.4E-02	1.6E-04	-7.3E-02	2.8E-02	9.4E-03
	10	rs2935713	123432188	A	-4.4E-02	1.3E-02	6.0E-04	-6.0E-02	2.4E-02	1.2E-02

Group1: individuals without any conditions of hypertension, diabetes or osteoporosis

Group2: individuals with hypertension, diabetes or osteoporosis

Beta: Regression coefficient (unit, mg/dl)

SE: Standard error of Beta

Supplementary Table 5. Details of the discovery and replication cohorts.

Cohort	Serum level (mg/dl)		Age (year)		Gender (Male/Female)
	Mean	SD	Mean	SE	
Discovery	1.92	0.2	8.6	6.3	1233/1023
Replication	1.92	0.2	11.1	4.6	108/125
